# Estimating Sustainable Live-Coral Harvest at Kamiali Wildlife Management Area, Papua New Guinea

**DOI:** 10.1371/journal.pone.0140026

**Published:** 2015-10-05

**Authors:** Ken Longenecker, Holly Bolick, Ross Langston

**Affiliations:** 1 Department of Natural Sciences, Bishop Museum, Honolulu, Hawaiʻi, United States of America; 2 Department of Natural Sciences, Windward Community College, Kāneʻohe, Hawaiʻi, United States of America; National Taiwan Ocean University, TAIWAN

## Abstract

Live coral is harvested throughout the Indo-West Pacific to make lime, used in the consumption of the world’s fourth-most consumed drug, betel nut. Coral harvesting is an environmental concern; however, because lime-making is one of the few sources of income in some areas of Papua New Guinea (PNG), the practice is unlikely to stop. To better manage coral harvest, we used standard fishery-yield methods to generate sustainable-harvest guidelines for corymbose *Acropora* species found on the reef flat and crest at Lababia, PNG. We constructed a yield curve (weight-specific net annual-dry-weight production) by: 1) describing the allometric relationship between colony size and dry weight, and using that relationship to estimate the dry weight of *Acropora* colonies *in situ*; 2) estimating annual growth of *Acropora* colonies by estimating *in situ*, and describing the relationship between, colony dry weight at the beginning and end of one year; and 3) conducting belt-transect surveys to describe weight-frequencies and ultimately to predict annual weight change per square meter for each weight class. Reef habitat covers a total 2,467,550 m^2^ at Lababia and produces an estimated 248,397 kg/y (dry weight) of corymbose *Acropora*, of which 203,897 kg is produced on the reef flat/crest. We conservatively estimate that 30,706.6 kg of whole, dry, corymbose, *Acropora* can be sustainably harvested from the reef flat/crest habitat each year provided each culled colony weighs at least 1805 g when dry (or is at least 46 cm along its major axis). Artisanal lime-makers convert 24.8% of whole-colony weight into marketable lime, thus we estimate 7615.2 g of lime can be sustainably produced annually from corymbose *Acropora*. This value incorporates several safety margins, and should lead to proper management of live coral harvest. Importantly, the guideline recognizes village rights to exploit its marine resources, is consistent with village needs for income, and balances an equally strong village desire to conserve its marine resources for future generations.

## Introduction

Lime, including calcium oxide (*i*.*e*., CaO) and calcium hydroxide (*i*.*e*., Ca(OH)_2_) manufactured from live coral is commonly used as an adjuvant for the world’s fourth-most consumed drug, *Areca catechu*. Only nicotine, ethanol, and caffeine are more-commonly used [1]. An estimated 600 million people [1,2] chew some combination of the drupe of the palm *A*. *catechu*, lime powder, and part of the betel plant *Piper betle* for a sense of well-being, heightened alertness, increased stamina, and as an anorectic [3]. Because it is most-frequently chewed with the betel plant and it is popularly called a nut, betel nut is the English name for the areca drupe [2]. In Papua New Guinea, it is called *buai*.

The geographic range of betel nut use can roughly be described as the countries bordering or contained within the Western and Central Indo-Pacific (see [2, 4] for lists and limits). In coastal areas, betel-nut lime is produced by heating corals or mollusc shells [2]. In Papua New Guinea, live *Acropora* species are the preferred source of lime because a purer white powder can be produced relative to other sources [5]. On Andra Island, PNG, lime production consumes approximately 2100 m^3^ of live *Acropora* per year [5].

Coral harvesting may dramatically alter coral reefs and their associated communities. Coral mining for construction materials, including lime production, has been classified as one of the main, local, anthropogenic threats to coral reefs [6]. At Mafia Island, Tanzania, where an estimated 2496 m^3^ of live coral is removed annually, mined sites had reduced live coral cover and lower fish abundance and diversity relative to un-mined sites [7]. In the Maldives, fish community structure at mined sites was clearly different from that at un-mined sites [8]. Coastal communities in Papua New Guinea, many of which depend on subsistence fishing for their primary source of dietary protein, recognize that betel-nut lime production may threaten their well-being; coral harvesting was the most frequently mentioned threat to coral reefs and second-most frequently mentioned threat to fishery resources by residents of Andra Island [5].

Conflicting with these environmental concerns are immediate economic needs. For instance, residents of Lababia (Morobe Province, Papua New Guinea) live a predominantly subsistence lifestyle. However, they need a modest income for medicine and school fees; the average person contracts malaria about twice a year and primary education is not free [9]. In coastal Morobe Province, lime production is the fifth most-important source of household income, and in Lababia it is the fourth-most-frequent income generating activity [9]. In the neighboring village of Salus, 100% of households engage in lime production [10].

**Fig 1:**
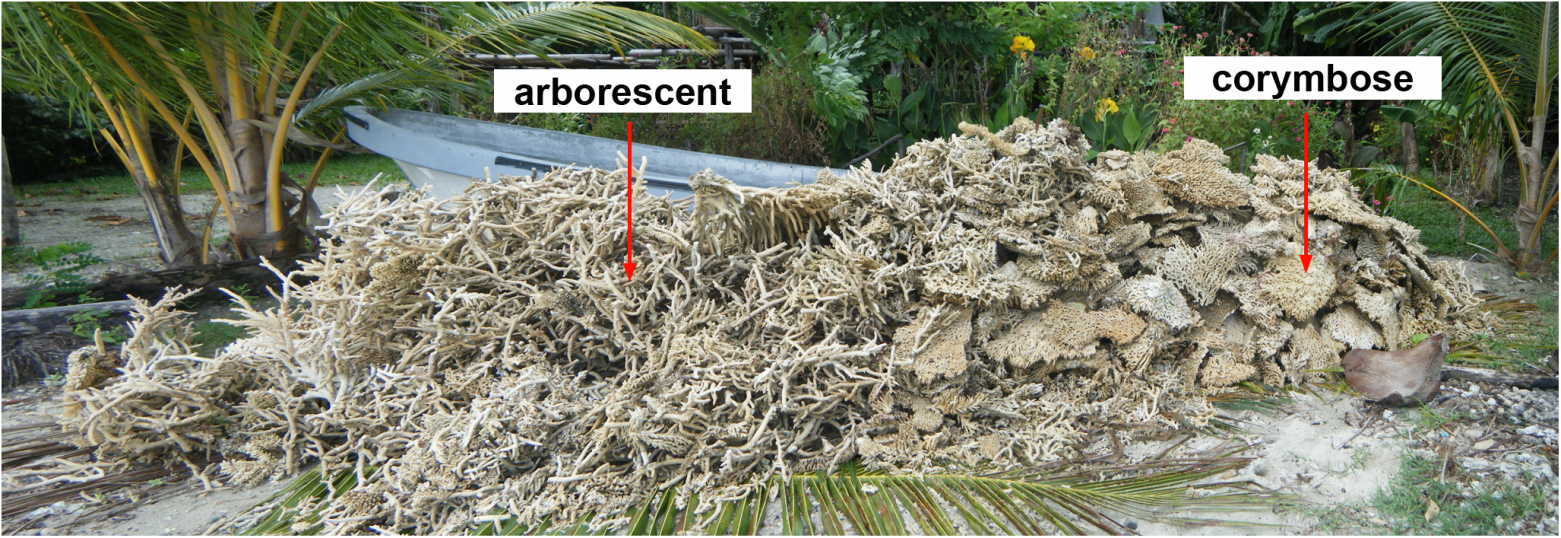
Corals on Lababia Island, PNG harvested for betel-nut lime production. We found this pile a few hours after a local informant told us the community ceased harvesting coral. We learned that harvest is frequent enough for collectors to know how much lime will be produced every time the boat in the background is filled to the gunwales. Photo: K. Longenecker.

Since 2006, the land and water under the customary tenure of Lababia has been the focus of a conservation initiative attracting researchers from developed countries to the area [11]. Some of these well-meaning individuals expressed disapproval of the live-coral harvesting, stating (without location-specific evidence) that the practice was unsustainable. This approach did not have the intended effect of halting live-coral harvest because residents still needed the revenue from lime production. However, residents feared upsetting researchers and thus losing the income derived from research projects, so coral harvesting became a covert activity (Fig 1). The sociocultural framework of PNG makes it more likely that successful conservation interventions will result from changing harvest practices (if necessary), not from attempting to stop the rights of villages to harvest their resources [5].

Here, we take a fishery approach to model annual production of acroporid corals on reefs under the customary tenure of Lababia village. Our ultimate goal is to present guidelines for the sustainable harvest of whole coral colonies. However, because the village does not possess high-capacity scales necessary to weigh coral, we convert our guidelines into a more-practical metric: weight of marketable lime. These estimates will give scientific advisors and resource owners, respectively, the information necessary to evaluate the sustainability of local coral-harvesting practices.

Our observations suggest *Acropora* species form nearly 100% of the harvest; those with an arborescent (branching) morphology represent half of the volume collected and the other half is composed of corymbose (plate- or table-like) forms (see Fig 1). Here we focus on the latter. *Acropora* taxonomy is complicated, and the corymbose forms at our study site represent at least three species; however, local folk taxonomy recognizes all corymbose *Acropora* as the same type. From a functional standpoint, this morphological grouping is the taxonomic resolution needed to evaluate the sustainability of half of the live-coral harvest at Lababia.

## Methods

### Ethics Statements

Our study site is collectively owned by residents of Lababia, a subset of whom form the Kamiali Wildlife Management Committee (KWMC). Approval for field work was granted at the local level by the KWMC and at the national level during the process of obtaining PNG research visas. We did not work on vertebrate animals, nor were protected species were sampled.

### Study Site

Lababia (7.287965°S; 147.123581°E) is located on the Huon Coast of Papua New Guinea, approximately 64 km SSE of the port city, Lae. Approximately 600 residents hold traditional tenure over their natural resources. In 1996, they established the Kamiali Wildlife Management Area (KWMA), with 32,000 hectares of terrestrial habitat and 15,000 hectares of adjacent marine habitat. Lababia Village is concentrated along the northern portion of KWMA, where the shoreline is exclusively sandy beach. The southern shoreline is dominated by fringing reefs on Capes Dinga and Roon. Fringing reefs also surround the islands of Lababia and Jawani. The seaward portions of reef flats typically feature carbonate bench or coral beds with occasional patches of sand or rubble. The reef crest features a high abundance and diversity of corals, although occasional beds of coral rubble also occur. The fore reef is steep, typically descending 20 to 30 m, and features corals, consolidated carbonate substrate, and rubble. At the base, the fore reef gives way to sediments to depths of about 100 m and covering most of the marine area. Some coral outcroppings, patch reefs and pinnacles are interspersed throughout this sedimentary area.

### Size-Weight Relationship

**Fig 2:**
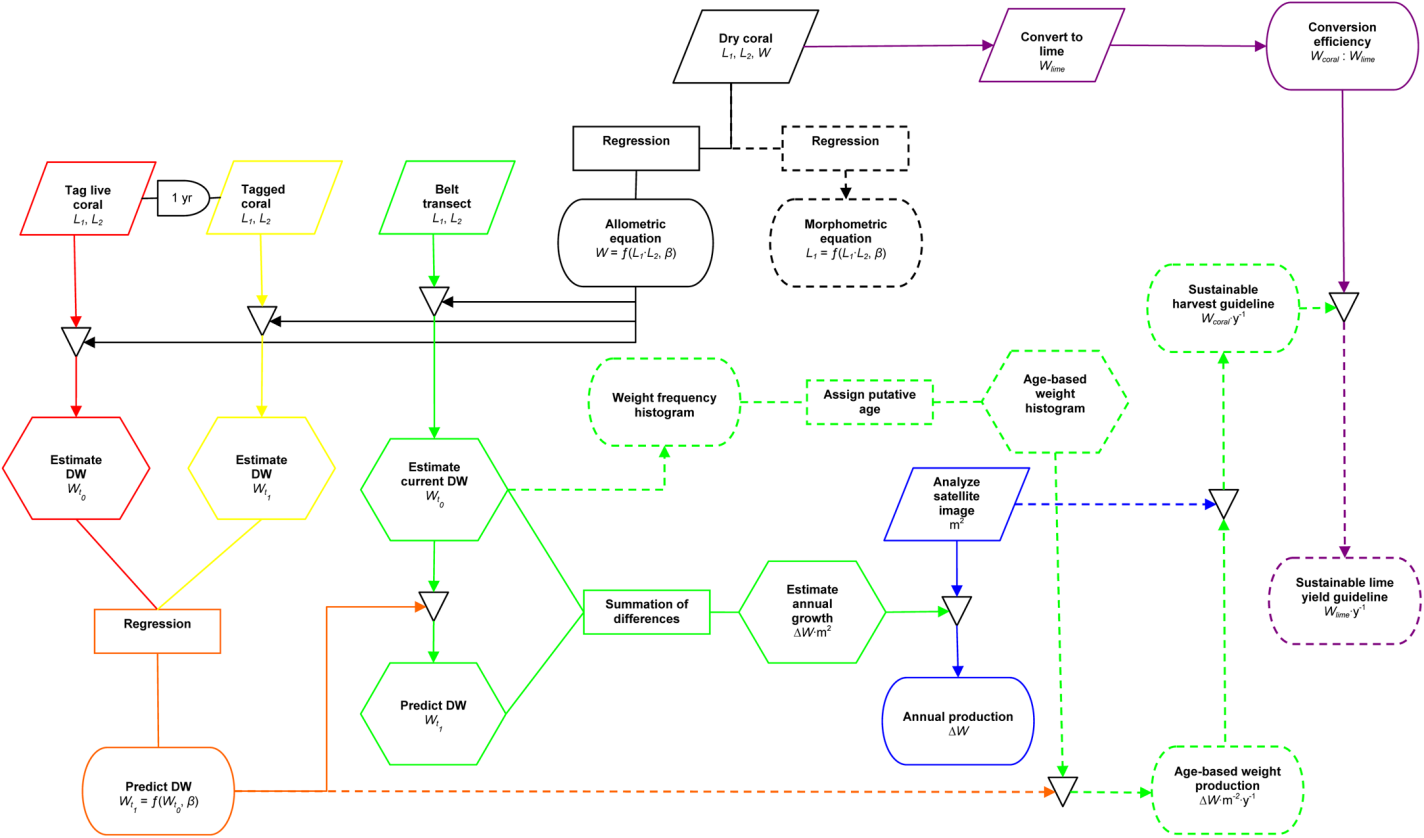
Work flow to estimate sustainable lime production at KWMA. Parallelograms represent data gathering, rectancles represent mathematical processes, arches represent delays, hexagons represent intermediate estimations or predictions, stadiums represent results presented herein, triangles represent the application of intermediate results to generate subsequent estimates.

**Fig 3:**
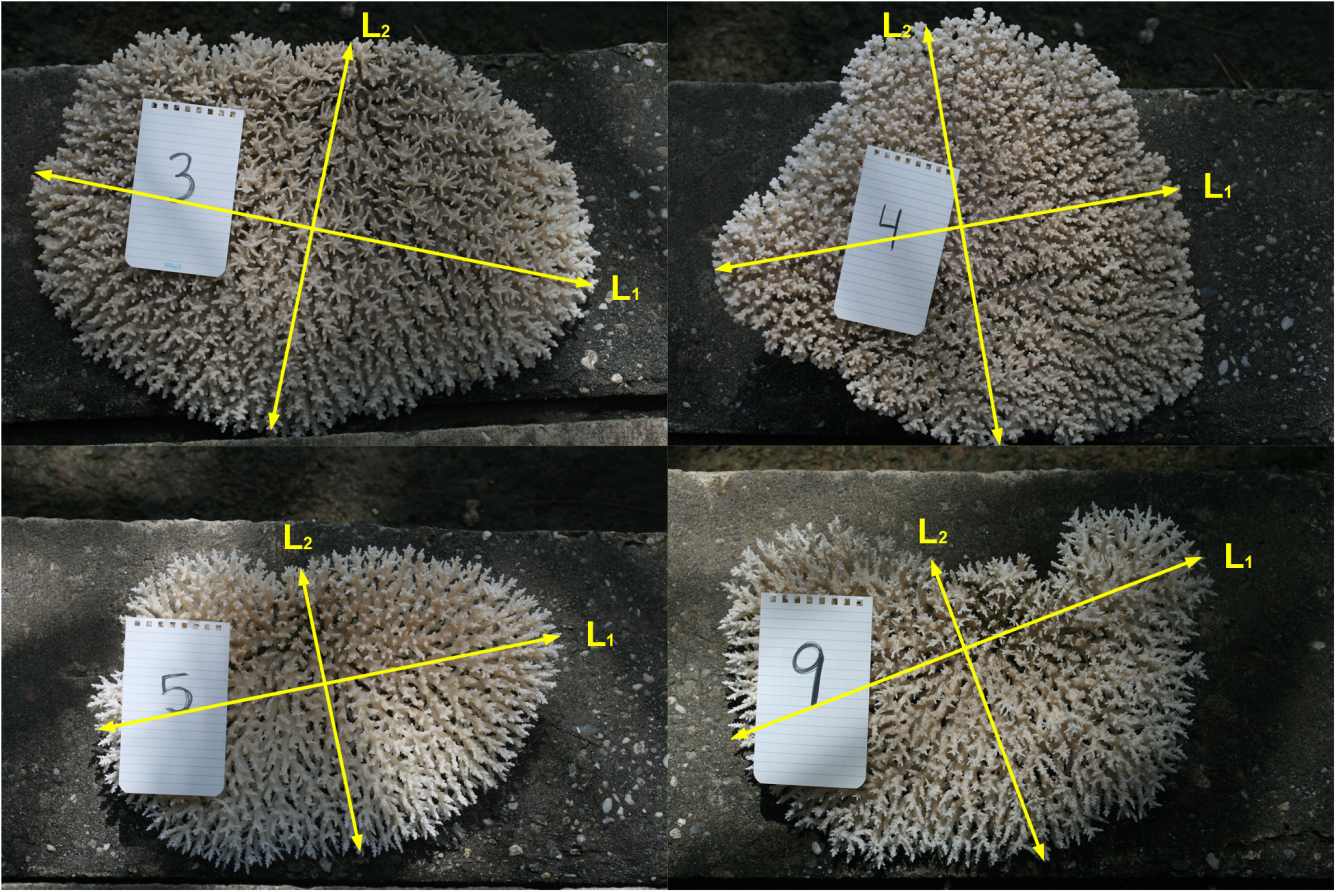
Major (*L*_*1*_) and minor (*L*_*2*_) axes. These were measured to construct the allomentric relationship and to estimate *in situ* dry weight of corymbose *Acropora* colonies at KWMA. Photos: K. Longenecker.

To enable us to predict, *in situ*, the dry weight of corymbose *Acropora* colonies (Fig 2, solid black portion), we opportunistically collected a broad size-range of whole specimens from 1–3 June 2011 and from 18–22 February 2013. Specimens were air-dried until 10 June 2011 (7–9 days) and 2 June 2013 (100–104 days), respectively. For each specimen, we measured to the nearest cm the major axis of the plate (*L*_*1*_) and a perpendicular axis (*L*_*2*_) of the plate at the approximate mid-point of the long axis (Fig 3). We then removed any adherent reef substrate from the base of the specimen, and weighed the specimen on a hanging spring scale.

We then determined the body-size variable that best predicted dry weight (*W*) in the allometric relationship, *W* = *aS* ^*b*^, where *S* is body size. We performed linear regression analysis on log_10_-transformed *S* and *W* data to estimate allometric parameters where the intercept equals log_10_(*a*) and the slope equals *b*.

### Annual-Growth Predictors

To estimate the annual weight change of individual corymbose *Acropora* colonies, we used nylon cable ties to attach pre-numbered aluminum racetrack tags to haphazardly chosen corals with 100% tissue covering (Fig 2, red portion). From 27 May through 8 June 2011, we tagged a total 133 colonies of varying sizes throughout their observed depth range (to ~25 m) at six sites scattered throughout KWMA. Upon tagging, we measured the axes *L*_*1*_ and *L*_*2*_, and noted depth and reef zone (reef flat, reef crest, fore reef). We returned to the tagged colonies 25–29 May 2012 and measured (as above) only the portion of the colony covered by living tissue (Fig 2, yellow portion). Colonies with 100% tissue mortality were given a value of zero for each dimension. Colonies that could not be relocated were excluded from further analysis (*i*.*e*., because we did not know their fate, we made no assumptions about their growth). However, colonies with known partial or total mortality were included in the analysis. We used the allometric relationship (Fig 2, solid black portion) to estimate the weight of each colony in 2011 (*t*_0_) and 2012 (*t*_1_). We used regression analysis to construct reef-zone-specific equations to predict final weight $\left(W_{t_{1}}\right)$ as a function of the original weight $\left(W_{t_{0}}\right)$ of corymbose *Acropora* colonies (Fig 2, orange portion). In subsequent calculations, we assume the time between coral measurements was one year. Thus the resulting regression equations are annual-growth predictors.

### Density, Size Structure, and Annual-Growth Estimates

To describe the density and size structure of corymbose *Acropora* colonies at KWMA, we surveyed 10 m^2^ belt transects from 30 May–3 June 2012 and from 3–6 June 2013 (Fig 2, green portion). At eight sites scattered throughout KWMA, we deployed along several isobaths within each reef zone a 10-m fiberglass measuring tape, noted depth and zone, and measured all corymbose *Acropora* colonies whose centers fell within ½ m of the tape. As above, we measured the axes *L*_*1*_ and *L*_*2*_. Beginning at 21 m and working progressively shallower, transects were distributed uniformly up the fore reef and reef crest (averaging every 1.5 m), then in approximately equal distances across the reef flat. Because the depth and extent of each zone varied by location, the number of transects in each zone also varied. We used the allometric relationship (Fig 2, solid black portion) to estimate the original weight of each colony in each transect $\left(W_{t_{0}}\right)$, and used these data to construct reef-zone-specific weight histograms (Fig 2, dashed green portion).

To estimate annual weight change per square meter for each transect, (Fig 2, solid green portion) we applied the appropriate reef-zone-specific annual-growth equation (Fig 2, orange portion) to predict the weight of each colony in one year $\left(W_{t_{1}}\right)$. Thus, for each transect, annual weight change m^−2^ (Δ*W*) was predicted by the equation:

$$\Delta W={\sum_{i=1}^{n}{\left(W_{t_{1}}-W_{t_{0}}\right)}_{i}\cdot 10\text{m}}^{-2}$$

where *n* is the number of corymbose *Acropora* colonies in a given transect.

### Reef Area

To estimate total reef area at KWMA, we used ImageJ to analyze a GeoEye–1 satellite image with 0.5 meter resolution (Fig 2, blue portion). We assumed reef crests and flats were horizontal surfaces, and calculated the cumulative area occupied by each habitat. To estimate fore-reef area, we estimated the cumulative distance covered by the seaward edge of all reef crests. We then assumed that all fore-reef habitat dropped vertically from a reef crest at 0 m to a bottom at 24.38 m (the lowest depth we observed corymbose *Acropora* at KWMA), thus our estimate of total fore-reef area is equal to reef-crest distance multiplied by 24.38 m. Jawani Island (7.341407°S, 147.207784°E) is included within the formal boundaries of KWMA. However, whether Lababia residents have rights to the marine resources surrounding the island is unclear. We conservatively excluded the reef surrounding Jawani Island from our area estimates.

### Annual Production and Sustainable Harvest Estimates

We estimated total annual weight change of corymbose *Acropora* at KWMA by multiplying the total area in each reef zone (Fig 2, blue portion) by the mean Δ*W* (predicted annual weight change m^−2^) value for each reef zone (Fig 2, solid green portion). We then summed the products for all zones.

To develop a sustainable-harvest recommendation, we estimated weight-specific annual net production (as dry weight). Residents do not SCUBA dive at KWMA, therefore we assumed coral harvesting is limited to the reef-flat and -crest habitats and generated a sustainable harvest estimate on the basis of colonies from these habitats only (*i*.*e*., colonies from the slope habitat are not included in production modeling).

We grouped weight by putative age (Fig 2, dashed green portion), in whole years, by first assuming that all colonies with axis lengths ≤ 8 cm were ≤ one-year-old (*Acropora nasuta* in Okinawa are immature when ≤ 8 cm in diameter [12]; the outer 4 cm of a colony is not reproductive in 11 *Acropora* species from Kenya [13]). We defined the upper limit of all subsequent weight classes as the sum of the upper limit (max*W*) of the previous weight class plus the expected annual dry-weight produced (Δ*W*′) by the previous weight class $\left(\max W_{t+1}=\max W_{t}+\Delta W_{t}^{\prime }\right)$. We estimated Δ*W*′ (Fig 2, orange portion) by multiplying the appropriate reef-zone-specific estimate of percent-weight-change per year (*b* − 1, where *b* equals the slope of the appropriate annual-growth-predictor equation) by the midpoint of each weight class.

We then constructed a yield curve (weight-specific net annual-dry-weight production) from belt-transect data (Fig 2, dashed green portion). As above, we used the allometric relationship (Fig 2, solid black portion) and the annual-growth-predictor equation (Fig 2, orange portion) to estimate $W_{t_{0}}$ and $W_{t_{1}}$, respectively, for each colony. We used $W_{t_{0}}$ to assign each colony to a weight class. For each weight class, annual production (AP) was estimated by the equation:

$$\text{AP}=\sum_{i=1}^{n}{\left(W_{t_{1}}-W_{t_{0}}\right)}_{i}\cdot A^{-1}$$

where *n* is the number of colonies in a given weight class, and *A* is the total area surveyed.

The peak of the yield curve represents the size at which maximum yield occurs (if we assume our growth estimators are accurate, and the observed size structure represents an unfished population in a steady state). In theory, all individuals larger than this size class can be harvested without negatively impacting the abundance and production of smaller size classes. In the interest of generating a more-conservative harvest recommendation, we suggest a harvest equivalent to the estimated annual net dry-weight production of the next-larger weight class. We obtained that estimate from the product of the estimated annual production by that weight class (Fig 2, dashed green portion) and the total area occupied by reef-flat and-crest at KWMA (Fig 2, blue portion).

### Harvestable Size

Our guidelines suggest the minimum dry weight a colony should attain before being harvested. Because dry weight would be understandably difficult for a collector to estimate for colonies *in situ*, we converted the dry-weight guideline to minimum major-axis length (*L*_*1*_). Here, we used the allometric relationship to estimate the (*L*_*1*_ ∙ *L*_*2*_) value, in whole numbers, that would result in minimum harvestable dry weight. We then used the statistical methods described above (Size-Weight Relationship) to describe the morphometric relationship (Fig 2, dashed black portion) between *L*_*1*_ and (*L*_*1*_ ∙ *L*_*2*_) for all untagged colonies measured during this study (*i*.*e*., those used to generate the allometric relationship, those measured prior to tagging, and those encountered during belt-transect surveys). We used the morphometric relationship to estimate the minimum *L*_*1*_ that would result in the (*L*_*1*_ ∙ *L*_*2*_) value necessary for a colony to have attained minimum harvestable dry weight.

### Lime Conversion Efficiency

To determine coral-to-lime conversion efficiency (Fig 2, purple portion), we delivered to community lime-making artisans the 36 dried, measured, and weighed colonies collected in 2013 to generate the allometric relationship (see Size-Weight Relationship). Using only the corals we provided, the artisans made lime using their standard procedure, which has been documented in a short video [14]. A portion of each colony was not used because it was judged by the artisans to be unsuitable for lime making. We determined the cumulative weight of the discarded (*i*.*e*., unprocessed) coral and of marketable lime. We then determined conversion efficiency on the basis of total weight of coral delivered and for the total weight of coral processed.

### Sensitivity Analysis

We examined the influence of the various intermediate estimates (*e*.*g*., allometric relationship, annual growth predictors, reef area, and coral-to-lime conversion efficiency) on our final sustainable lime production estimate. We reduced by 10% a model parameter (allometric relationship and annual growth predictor) or intermediate variable (reef area and conversion efficiency), re-calculated sustainable lime production, and determined the percentage change from our original sustainable lime production estimate.

## Results

**Fig 4:**
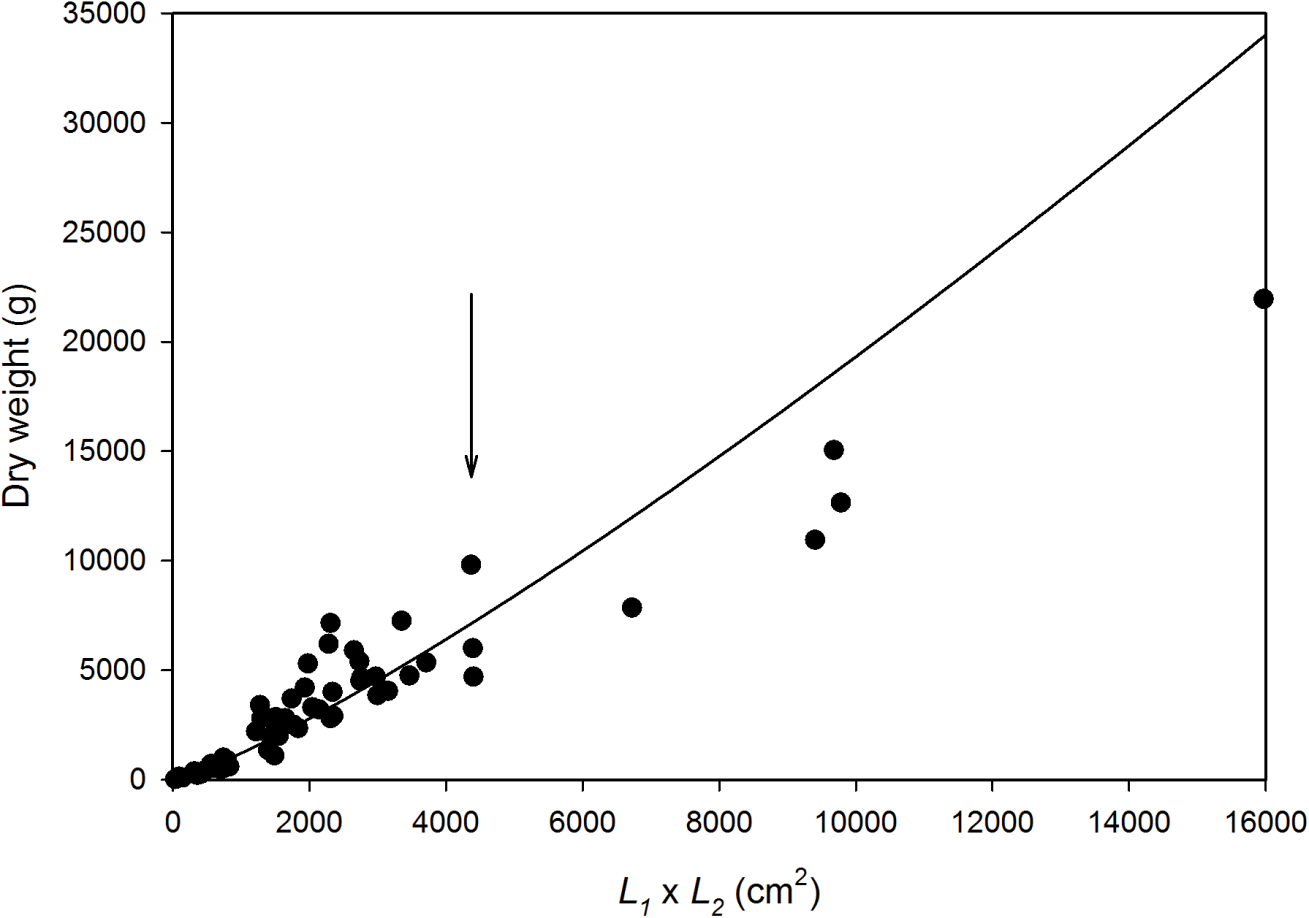
*In situ* dry-weight predictor for corymbose *Acropora* colonies at KWMA. *W* = 0.2951(*L*_*1*_ ∙ *L*_*2*_)^1.2040^; r^2^ = 0.936. 99% of colonies to which we applied the relationship had area estimates ≤ 4368 cm^2^ (arrow).

On the basis of measurements of 60 colonies, a simple metric was the best predictor of the dry weight (g) of corymbose *Acropora* colonies at KWMA (Fig 4). The product of plate-axis measurements *L*_*1*_ and *L*_*2*_, in cm, was a better dry-weight predictor than either axis alone. The allometric relationship (Fig 2, solid black portion) we used to predict the dry weight of *in situ* colonies is: *W* = 0.2951(*L*_*1*_ ∙ *L*_*2*_)^1.2040^ (r^2^ = 0.936). The equation we chose appears to overestimate weight for the largest colonies. However, because 99% of the colonies for which we estimated *W* had (*L*_*1*_ ∙ *L*_*2*_) measurements ≤ 4368 cm^2^, the relationship appears to be suitably descriptive for the overwhelming majority of our observations (Fig 4). This relationship is also satisfying on a theoretical basis because the shape of corymbose *Acropora* is roughly elliptical and the equation for the area of ellipse $\left(L_{1}\cdot L_{2}\cdot \frac{\pi }{4}\right)$ is a function of the size metric we used. As expected, an allometric relationship using elliptical area as the independent variable had the same *r* ^*2*^ and *b* values as the relationship we chose; however, it resulted in larger overestimates of weight for the largest colonies.

We tagged a total 133 colonies in 2011 (Fig 2, red portion) and were able to find 61 (45.8%) of the tagged corals after approximately one year (Fig 2, yellow portion). The weighted mean time between weight estimates was 359.8 days, or slightly less than one year.

We do not know the fate of the lost colonies; however, we did find three loose tags, one tag completely enveloped by new coral growth, and two dislodged colonies. Tags may have been lost or overlooked or colonies may have been transported off the reef (by waves, currents, or harvesting). A t-test (two sample, equal variances) comparing original (2011) colony size of re-located versus lost tags was not significant, suggesting that size-dependent factors (*e*.*g*., overlooking small colonies or large colonies being transported off the reef) did not influence tag recovery.

Of the colonies we did re-locate, 31.1% experienced partial or complete mortality. Casual observation suggested that the incidence of mortality for tagged colonies was not different from that of nearby untagged colonies.

**Fig 5:**
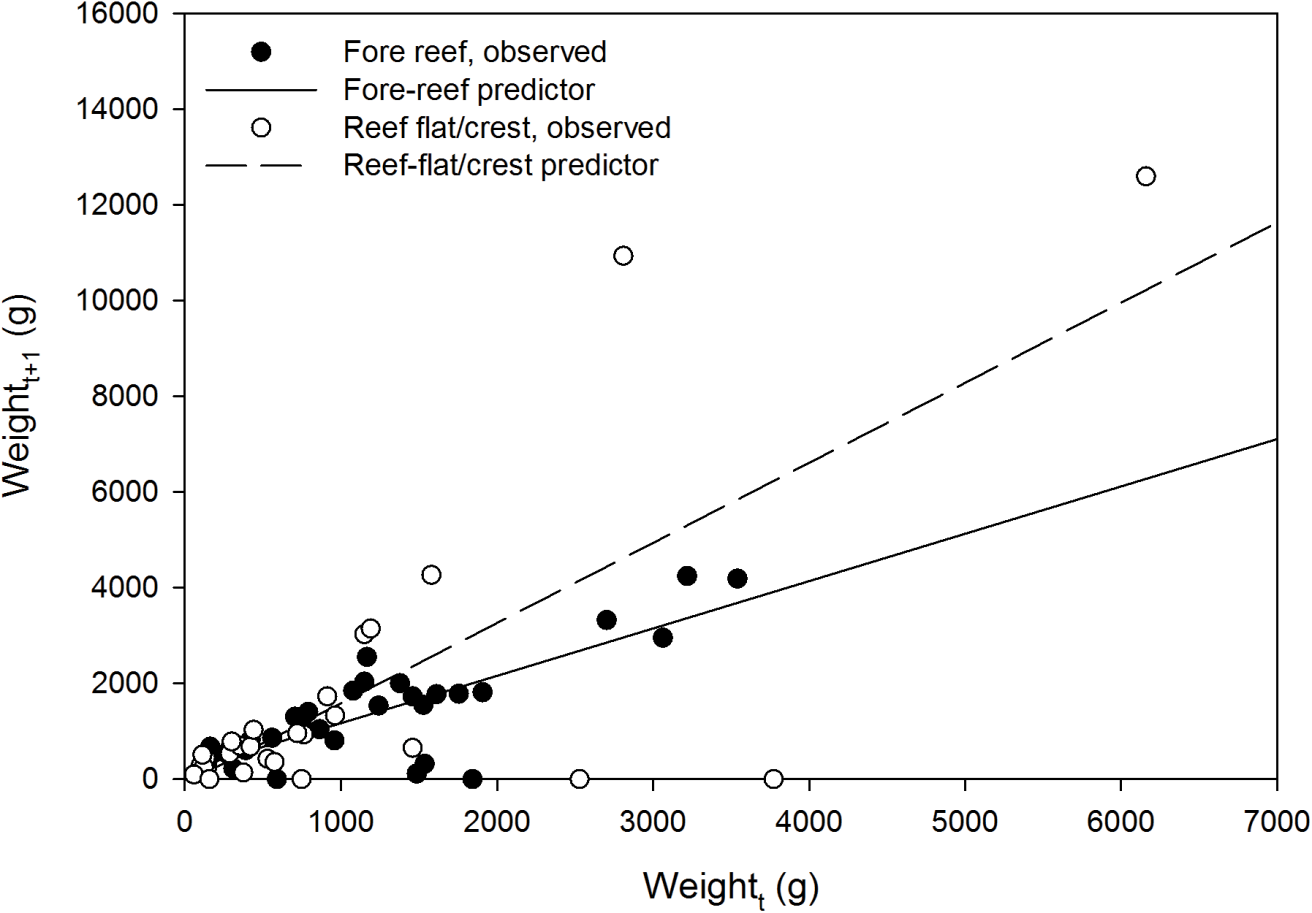
Estimated weight of tagged colonies at the beginning (*W*_*t*_) versus end (*W*_*t+1*_) of one year. Reef flat/crest: *W*_*t+1*_ = 1.67(*W*_*t*_) − 66.49; r^2^ = 0.549. Fore reef: *W*_*t+1*_ = 0.990(*W*_*t*_) + 140.57; r^2^ = 0.668.

Exploratory data analysis suggested habitat-dependent growth. As a group, colonies on the reef flat and crest had faster mean but more-variable between-colony growth than colonies on the fore reef (Fig 5). Linear equations can be used to predict, on the basis of starting weight in g (*W*_*t*_), the weight of a corymbose *Acropora* colony after one year (*W*_*t+1*_) at KWMA (Fig 2, orange portion). For colonies on the reef flat and crest: *W*_*t+1*_ = 1.67(*W*_*t*_) − 85.26 (n = 28, r^2^ = 0.549); whereas on the fore reef: *W*_*t+1*_ = 0.99(*W*_*t*_) + 180.31 (n = 32, r^2^ = 0.648).

**Fig 6:**
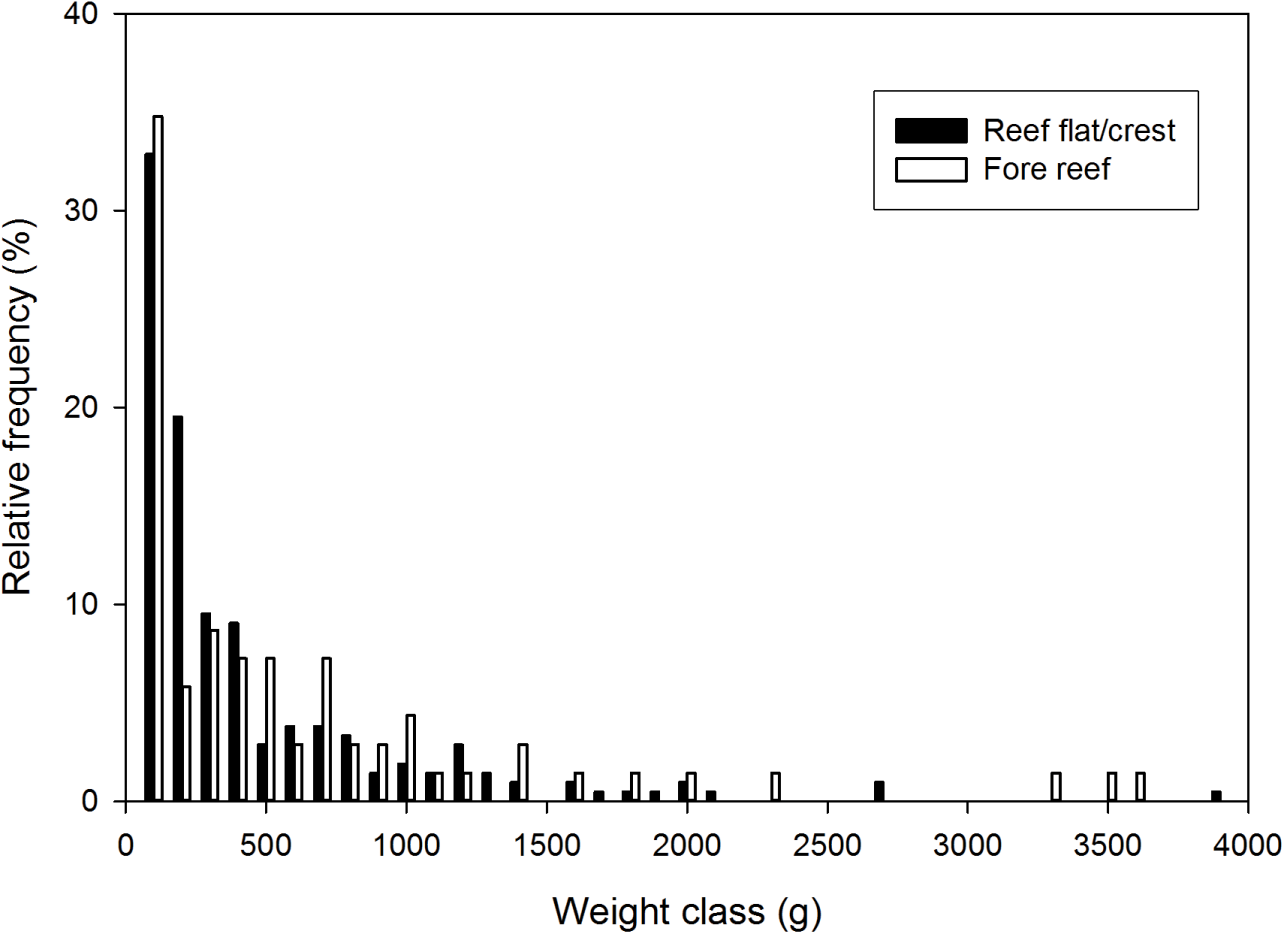
Relative frequency plot of estimated weights of corymbose *Acropora* colonies at KWMA. 70 fore reef specimens, 212 reef flat/crest specimens. Colonies with likely overestimated weights are not shown (8059 and 18,877 g colonies from the reef flat/crest, and a 26,396 g colony from the fore reef).

We surveyed a total of seventy 10-m^2^ transects (Fig 2, green parallelogram). Fifty-three transects were in the reef flat/crest habitat, whereas 17 were in the fore reef habitat. Fig 6 shows the distribution of estimated colony weights (*W*_*t*_) in each habitat. Colony density was not significantly different between the two habitats (0.400 ± 0.365/m^2^ on the reef flat/crest and 0.412 ± 0.473/m^2^ on the fore reef, *t* = 0.107, df = 68, *P* = 0.915). Mean estimated dry weight per m^2^ was nearly twice as high on the fore reef (390.5 ± 646.1 g) than on the reef flat/crest (211.1 ± 366.7 g). However, the values were not significantly different (*t* = 1.090, df = 19, *P* = 0.145). Because of faster growth on the reef flat/crest (Fig 5), estimated mean annual dry weight increase per m^2^ (Fig 2, solid green portion) was higher there (110.6 ± 228.6 g/m^2^) than on the fore reef (70.3 ± 84.1 g/m^2^). However, the difference was not statistically significant (*t* = -1.074, df = 67, *P* = 0.143).

We estimate that there is a total 2,476,550 m^2^ of reef at KWMA within the depth range of corymbose *Acropora* colonies. Of this the reef flat/crest occupies 1,843,555 m^2^, nearly three times the area occupied by the fore reef (632,995 m^2^). Thus, we estimate that the reef flat/crest produces 203,897 kg of dry, corymbose *Acropora* skeleton per year, and that the fore reef produces 44,500 kg/y, for a total increase of 248,397 kg/y at KWMA (Fig 2, blue portion).

**Fig 7:**
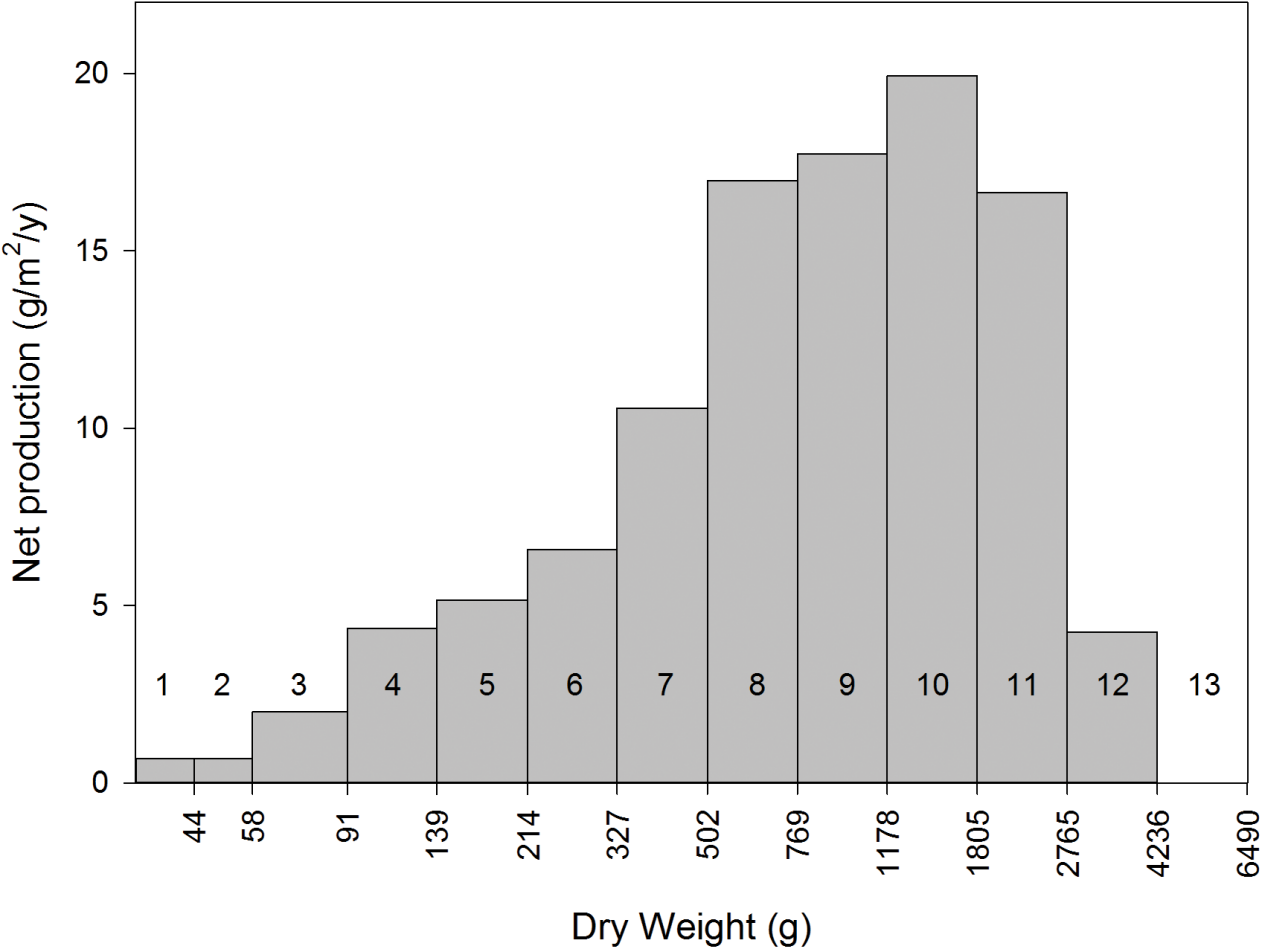
Net annual dry-weight production, by weight class, of corymbose *Acropora* on reef flats and crests at KWMA. Assuming weight and age are positively related, each bar represents a year-class. Putative age is indicated by the number above or in a given bar. Excluded from the plot is the influence of two colonies estimated to be 8059 and 18,877 g. Note that dry weight is plotted on a logarithmic scale.

We used the allometric relationship to estimate that the upper weight limit of colonies putatively less than one-year-old is 44.1 g. Our annual-growth-predictor equation for the reef flat/crest was not suitable for constructing putative 1-yr weight classes because it predicted that colonies < 51.1 g experience 100% mortality and that colonies < 127.3 g lose weight (*i*.*e*., the equation predicted negative growth for the smallest weight classes). We instead used a linear regression forced through the origin [*W*_*t+1*_ = 1.64(*W*_*t*_); *r* ^*2*^ = 0.548] to determine the limits of putative 1-yr weight classes (Fig 2, dashed green portion). The annual growth rate predicted by the revised regression was lower than that for the original annual-growth-predictor (64.4% versus 67.5% per year, respectively). The resulting yield curve is presented in Fig 7. Considering the parameters of our conservative harvest rules, no colony weighing less than 1805 g should be collected. We estimate that colonies in the 1805–2765 g weight class will produce 16.66 g/m^2^/yr and that, with a total 1,843,555 m^2^ of reef flat/crest at KWMA, the net dry-weight production of this weight class of corymbose *Acropora* colonies is 30,706.6 kg/y.

**Fig 8:**
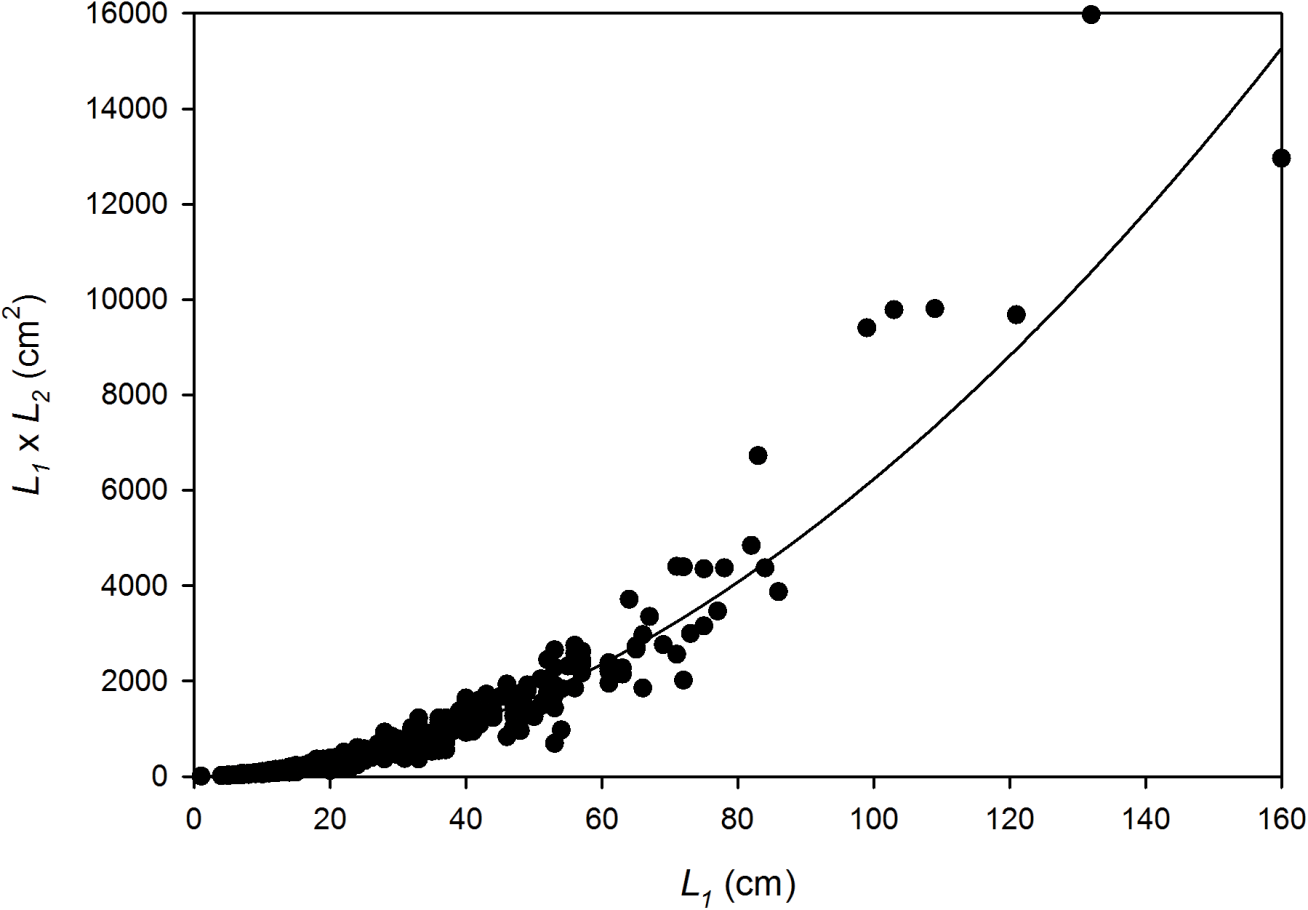
Harvestable-size estimate. (*L*_*1*_ ∙ *L*_*2*_) = 0.9601(*L*_*1*_)^1.9062^; *r* ^*2*^ = 0.970. Colonies with a major-axis length (*L*_*1*_) of 41 cm are predicted to have attained harvestable size.

The allometric relationship suggests colonies with an (*L*_*1*_ ∙ *L*_*2*_) value of 1397 will have attained 1805 g. The morphometric relationship (Fig 2, dashed black portion) between *L*_*1*_ and (*L*_*1*_ ∙ *L*_*2*_) is presented in Fig 8. The equation, (*L*_*1*_ ∙ *L*_*2*_) = 0.9601(*L*_*1*_)^1.9062^ (n = 475; *r* ^*2*^ = 0.970), predicts that colonies with a major-axis length (*L*_*1*_) of 46 cm will have an (*L*_*1*_ ∙ *L*_*2*_) value > 1397 and thus have attained harvestable weight.

The cumulative dry weight of corals delivered to the artisans was 199,400 g. Each colony had a major-axis length (*L*_*1*_) ≥ 42 cm. A total 52,900 g of coral were discarded prior to processing. The portions discarded were the central, basal region and the thicker area surrounding the basal region; the artisans judged these portions to be too dense to be processed into marketable lime. A total 49,450 g of lime was produced. Conversion efficiency based on whole-colony weight is 24.8%; whereas conversion efficiency for coral processed (*i*.*e*., ignoring the discarded portions) is 33.8% (Fig 2, solid purple portion). On the basis of whole-colony conversion efficiency, our results suggest the village can sustainably produce 7,615.2 kg of lime per yr (Fig 2, dashed purple portion).

Results of sensitivity analysis (Table 1) indicate that our sustainable lime production estimate is most influenced by the allometric relationship (Fig 2, solid black portion). A 10% reduction in the exponent of the allometric relationship resulted in a 70% reduction in our estimate. Least influential were our estimates of reef area (Fig 2, blue portion) and coral-to-lime conversion efficiency (Fig 2, solid purple portion). A 10% reduction in either of these led to a 10% reduction in our estimate.

**Table 1:** The influence of intermediate estimates on sustainable lime-production values. Parameter values were reduced by 10% to produce alternate, sustainable, annual lime-production estimates. Change is relative to our original estimate of 7,615.2 kg.

Intermediate Estimate	Parameter	Lime Production (kg)	Change (%)
Colony dry weight	*b* from allometric relationship	2287.6	-70
Colony weight change	Δ*W’* from alternate annual growth equation	4857.1	-36
Reef-flat and -crest area	*A*	6873.7	-10
Coral-to-lime conversion efficiency	%	6853.7	-10

## Discussion

Exploitation of live, stony corals is well-documented, and much concern has been expressed over the practice [5–8, 15–18]. Concomitantly, there have been very few suggestions that sustainable harvest is occurring or even a reasonable expectation (but see [19–21]). Socioeconomic conditions at Lababia, PNG make it unlikely that coral harvest for betel-nut lime production will stop within KWMA. Successful coral conservation in PNG will require changing patterns of exploitation rather than attempting to stop it [5]. Accordingly, we present life-history-based sustainable harvest guidelines for the corymbose *Acropora* community that represents approximately one-half of the coral colonies taken in KWMA.

Because we relied on empirical methods, our confidence in the sustainable-harvest guidelines we offer is directly related to sample size. One year after initial tagging, we located less than half the colonies used to estimate annual growth rates, raising the possibility that mortality is under-represented in our calculations. However, tags may have been just as likely to have been lost or overlooked. Because the incidence of mortality of the tagged colonies we did relocate did not seem to differ from that of nearby untagged colonies, we assume that our growth estimates are representative. Nonetheless, additional tagging efforts would likely produce more-robust estimates.

Another limitation of our study is that the annual production estimate is representative of conditions during a single year. Annual production estimates are influenced by abundance, growth rates, and mortality, all of which can be altered by biotic and abiotic factors that may change over time (*e*.*g*., predation and temperature). Accordingly, the results presented above should be considered tentative.

Given the preliminary nature of this study and our many assumptions, we are encouraged that the shapes of our size structure and yield curve match the general expectations of dynamic-pool models. If our assumption that age and weight are directly related is correct, the exponential decline of the weight histogram (Fig 6) is consistent with a steady-state recruitment and constant mortality [22]. If steady-state conditions prevail, the cumulative production of all cohorts present at a single time is the same as the production of a single cohort throughout its existence [23]. In the latter case, a cohort’s weight production will increase through time until losses to mortality exceed weight gain of the surviving individuals [23]. This describes our plot of annual net production (Fig 7) reasonably well.

We suggest it is sensible to apply our guidelines, even though tentative, to the *Acropora* community we studied. *Acropora* species are considered to be "weedy", thus their high recruitment and growth rates give surviving colonies a high capacity to replace harvested corals [21]. Further, in addition to generating a conservative harvest recommendation (the annual net dry-weight production of a single post-maximum-yield size class, rather than all individuals larger than the maximum-yield size class), our guidelines incorporate several potential safety margins. First, we assume that collection causes complete mortality of colonies. However, several lines of evidence suggest that collectors remove only the outer region of colonies and leave the thicker central and basal portions *in situ*: informants told us that corals are collected by breaking off the thinner portions of a colony; we observed only fragments fitting this description in village coral collections (Fig 1); and the lime-makers we commissioned to make lime from the whole colonies we provided discarded the thick, central and basal portions. Contrary to our assumption that collection causes complete mortality, the colony portions left *in situ* may survive and grow. Thus our production estimates may be low. A second potential safety margin is the large difference between putative size-at-reproduction and recommended harvest size at KWMA. Population replacement is encouraged when minimum-size limits are sufficiently larger than size-at-maturity [23]. Third, we suggest that the harvest can include any colony larger than the maximum size of the peak-production size class. This avoids concentrating exploitation mortality on a single size class, and helps avoid the possibility of creating an exploitation-induced production bottleneck. Fourth, our production estimates do not include the fringing reef around Jawani Island. Although it is included in KWMA boundaries, we received conflicting information about which village(s) and/or clan(s) has the right to its resources (we have never been able to gain access to the reef surrounding Jawani). If Lababia residents do control these resources, the approximately 148,291 m^2^ of reef flat/crest and 74,542 m^2^ of fore reef surrounding the island almost certainly represent additional production and reproductive output for KWMA. Such a non-harvested population reserve is another means of providing a reproductive cushion for an exploited population [23]. Finally, because collecting is depth-limited (SCUBA is not used at KWMA) the production estimates on which our harvest guidelines are based do not include the fore-reef population. An un-harvested deep-water population may serve as a reproductive reservoir for shallow-water recruitment, thus helping to insure sustainable yields [19]. However, we cautiously embrace this suggestion at KWMA because we are not sure that the deeper community has the same species composition as the shallower, exploited community. Nor are we certain that the deeper community is a significant source of new recruits. At nearby Salamaua, fecundity of *Acropora palifera* decreased as depth increased; the fecundity of colonies deeper than 15 m was one-fifth that of colonies shallower than 5 m, suggesting that deeper colonies may contribute few recruits to the population [24].

Sensitivity analysis shows that allometric relationship is the most influential intermediate estimate in our sustainable harvest calculation (Table 1), We have a high degree of confidence in this estimator because of the high coefficient of determination for the regression equation (r^2^ = 0.936). Further, we excluded from our sustainable harvest calculation the influence of colonies whose weights were likely to be overestimated by the allometric relationship (Figs 4 and 5). Thus, we feel justified in being less concerned about errors potentially resulting from the allometric relationship than we are about those resulting from the other intermediate estimates. Most influential of the latter is Δ*W’* from the alternate annual growth equation. We acknowledge, above, that additional tagging efforts would likely produce a more-robust growth equation. However, we emphasize that the possibility that this or either of the other two less-influential intermediate estimates (reef area or coral-to-lime conversion efficiency) are 10% too high is more than compensated for by our suggestion that harvest be restricted to the estimated annual production of the 1805–2765 weight class. Theoretically, sustainable harvest could include the annual production of all classes equal to or larger than that yielding peak production (*i*.*e*., the next smaller weight class: 1178–1805 g), or an additional 11,057 kg of lime.

An additional safety margin could be built into our harvest guidelines with adequate knowledge of the reproductive biology of this *Acropora* community. For instance on the Great Barrier Reef, more than 140 broadcast-spawning reef-coral species participate in multispecific, synchronous “mass spawning” events during a single week following the full moon in October or December [25]. For several *Acropora* species, oogenesis precedes spawning by 4–6 months [13, 26]. If a discrete spawning period occurs at KWMA, the production of new recruits could be encouraged by a harvest closure several months prior to spawning. However, spawning times vary widely for Indo-Pacific *Acropora* species. At any given time, *Acropora* is likely to be spawning in some part of the Indo-Pacific [13, 25, 27–31]. We simply do not know the reproductive season of corymbose *Acropora* species at KWMA. Further, there may not be a discrete season. *Acropora palifera* reproduces year-round near Lae, less than 60 km from KWMA [24], whereas further along the northern coastline of PNG, *A*. *formosa* and *A*. *hyacinthus* have a prolonged spawning at Madang [32]. An understanding of *Acropora* spawning patterns at KWMA would clarify whether a defined harvest season would help insure sustainability of the fishery.

We have suggested that the reef flat and crest habitat, combined, at KWMA can sustainably support the removal of 30,706.6 kg per year of corymbose *Acropora* colonies. We emphasize that this guideline assumes only whole colonies are collected, and that each is has a major-axis length (*L*_*1*_) of at least 46 cm or weighs at least 1805 g when dry.

Weighing the large piles of coral collected by Lababia residents (Fig 1) would be unwieldy (especially given that there is no high-capacity scale in this subsistence community). However, producers do know the weight of lime packaged for sale. To make our guidelines more practical, we developed a conversion factor to estimate the quantity of lime produced by a known dry weight of whole coral colonies. Our results indicate that artisanal lime production at Lababia is not very efficient. Empirical formulae indicate that, at 100% efficiency, the dry-coral:lime conversion is 1.8:1 [16]. However, we observed a 4.03:1 conversion efficiency for whole colonies and a 2.96:1 conversion efficiency when only the colony portions used for lime manufacture are considered. Assuming our single observation of lime-making produced a representative conversion efficiency, Lababia can sustainably produce 7,615.2 kg of lime each year from corymbose *Acropora* colonies, provided each harvested colony is at least 46 cm in its greatest dimension.

Because fishery statistics do not exist for PNG’s coral harvest, and because village residents do not maintain lime production records, we have an imperfect understanding of current levels of coral harvest and lime production at KWMA. We used published socioeconomic information [9–11, 33] to generate a rough estimate of annual lime production. Table 2 shows the step-by-step process we used to estimate that Lababia residents produce 3111.11 kg of lime per year from corymbose *Acropora* colonies. Current production appears to be less than half of our sustainable production estimate. Given the uncertainty of the current-production estimate, largely because much of the socioeconomic information is not Lababia-specific, we are reluctant to recommend an expansion of the corymbose *Acropora* fishery. Rather, we suggest that village lime makers should, initially, maintain current production levels and carefully record their output. The village can then compare current-production records to our sustainable-production recommendations to determine whether the fishery can bear expansion or should be scaled back.

**Table 2:** Derivation of an estimate of recent annual lime production at KWMA.

Estimate	Value	Unit	Geographic Range Represented	Source	Village-level Estimate	Unit
Village population	600	people	Lababia	[11]		
Average household size	5.4	people/household	Lababia & Salus	[9]	111.11	households
Average household income	1000	kina/year	Lababia	[33]	111,111.11	kina/year
Income from lime	1.12	% of total	Morobe Province	[9]	12,444.44	kina/year
Price of lime	20	kina/bag	Morobe Province	[10]	622.22	bags/year
Package size	10	kg/bag	Morobe Province	[10], present study	6222.22	kg/year
Corymbose *Acropora* harvest	50	% of total	Lababia	present study	**3111.11**	**kg/year**

We suggest that, in places such as Lababia where the immediate need for income may outweigh a village’s collective desire for long-term coral-reef conservation, life-history-based sustainable-harvest guidelines are more likely, than simply discouraging coral harvest, to result in desirable conservation outcomes. The results presented above are preliminary sustainable-harvest guidelines for corals representing approximately one-half of the betel-nut lime production at KWMA. We emphasize that these guidelines are limited to corymbose *Acropora* colonies at KWMA. Although our methods are applicable to other areas, our results may not be. Further, the same harvest limits may not apply to the arborescent *Acropora* that forms the other half of coral harvest for betel-nut lime production at KWMA.

We think the methods described herein can and should be applied to other species and in other locations. The methods are simple, inexpensive, and fast. A full study can be completed in one year, with only about one week of field work at the beginning and end of that year. In other words, these methods would be appropriate for research in developing countries where coral harvesting is perceived to be a threat to corals and their associated biota.

## Supporting Information

S1 File Size-Weight Data. “Year” = year the specimen was collected; “L1 (cm)” = major axis length of specimen, in centimeters; “L2 (cm)” = minor axis length of specimen, in centimeters; “DW (g)” = dry weight of specimen, in grams.
(XLS)

S2 File Tagging Data. “Tag #” = specimen number; “L1-t0 (cm)” = major axis length of specimen, in centimeters, at start of year; “L2-t0 (cm)” = minor axis length of specimen, in centimeters, at start of year; “L1-t1 (cm)” = length of major axis of specimen, in centimeters, at end of year; “L2-t1 (cm)” = length of minor axis of specimen, in centimeters, at end of year; “Reef Zone” = habitat category (reef flat, reef crest, fore reef). Blank values in L1-t1 (cm) and L2-t1 (cm) indicate the colony was not found at the end of the year; zero values in the same columns indicate the colony was found, but that it was completely dead.
(XLS)

S3 File Transect Data. “Date” = date of survey; “Reef Zone” = habitat category (fore reef, reef flat/crest); “Transect” = transect number; “Specimen” = specimen number, nested within transect number; “L1 (cm)” = length of major axis of specimen, in centimeters; “L2 (cm) = length of minor axis of specimen, in centimeters. If no specimens were observed on a transect, “Specimen” value is “NONE” and “L1 (cm)” and “L2 (cm)” are blank.
(XLS)
